# Validation of the King’s Brief Interstitial Lung Disease questionnaire in Idiopathic Pulmonary Fibrosis

**DOI:** 10.1186/s12890-019-1018-0

**Published:** 2019-12-19

**Authors:** Thomas Skovhus Prior, Ole Hilberg, Saher Burhan Shaker, Jesper Rømhild Davidsen, Nils Hoyer, Surinder S. Birring, Elisabeth Bendstrup

**Affiliations:** 1 Department of Respiratory Diseases and Allergy, Aarhus University Hospital, Aarhus, Denmark; 2 Department of Respiratory Medicine, Vejle Hospital, Vejle, Denmark; 3 Department of Respiratory Medicine, Herlev and Gentofte Hospital, Copenhagen, Denmark; 4 Department of Respiratory Medicine, Odense University Hospital, Odense, Denmark; 5 Centre for Human & Applied Physiological Sciences, School of Basic & Medical Biosciences, Faculty of Life Sciences & Medicine, King’s College London, London, UK; 6 Department of Respiratory Medicine, King’s College Hospital, London, UK

**Keywords:** Idiopathic pulmonary fibrosis, IPF, Health-related quality of life, Quality of life, The King’s Brief Interstitial Lung Disease questionnaire, K-BILD, The St. Georges respiratory questionnaire, 6-min walk test, 6MWT, Danish

## Abstract

**Background:**

Health-related quality of life (HRQL) is impaired in patients with idiopathic pulmonary fibrosis (IPF). The King’s Brief Interstitial Lung Disease questionnaire (K-BILD) is a validated measure of HRQL, but no previous studies have focused on the validity of K-BILD in IPF. Moreover, the relationship between K-BILD and dyspnoea or the 6-min walk test (6MWT) has not been assessed. The aim of this study was to validate K-BILD in the largest cohort of patients with IPF to date and assess how K-BILD correlates to dyspnoea and 6MWT.

**Methods:**

Firstly, K-BILD was translated into Danish using validated translation procedures. Consecutive patients with IPF were recruited. At baseline, patients completed K-BILD, the IPF-specific version of St. Georges Respiratory Questionnaire, University of California, San Diego Shortness of Breath Questionnaire (SOBQ) Short Form-36, and pulmonary function tests and 6MWT were performed. After 14 days, K-BILD and Global Rating of Change Scales were completed. Internal consistency, concurrent validity, test-retest reliability and known groups validity were assessed. Analyses were also performed in subgroups of patients with different time since diagnosis.

**Results:**

At baseline, 150 patients with IPF completed the questionnaires, and 139 patients completed the questionnaires after 14 days. K-BILD had a high internal consistency (Cronbach’s α = 0.92). The concurrent validity was strong compared to SOBQ (*r* = − 0.66) and moderate compared to 6MWT (*r* = 0.43). Intraclass correlation coefficients (ICC = 0.91) and a Bland Altman plot demonstrated a good reliability. K-BILD was also able to discriminate between patients with different stages of disease (*p* < 0.002, Δscore > 7.4) and most results were comparable in patients with different time since diagnosis.

**Conclusion:**

K-BILD is a valid and reliable instrument in patients with IPF and in patients with different time since diagnosis. To a major extent, K-BILD scores reflected the impact of dyspnoea on HRQL and the impact of physical functional capacity measured by the 6MWT to a moderate degree. Compared to PFTs alone, K-BILD provides additional information on the burden of living with IPF, and importantly, K-BILD is simple to implement in both research and clinical contexts.

**Trial registration:**

Clinicaltrials.org (NCT02818712) on 30 June 2016.

## Background

Idiopathic pulmonary fibrosis (IPF) is a progressive, fibrotic interstitial lung disease (ILD) with short life expectancy [1]. As the disease progresses, health-related quality of life (HRQL) deteriorates due to dyspnoea, decreased exercise capacity, loss of mental well-being and social isolation [2]. To address this issue, disease-specific HRQL questionnaires focusing on the main symptoms and life conditions in patients with IPF are warranted.

HRQL in patients with IPF has often been measured using St. George’s Respiratory Questionnaire (SGRQ), a disease-specific instrument developed for chronic obstructive lung disease (COPD) and asthma [3, 4]. However, ILD-specific HRQL questionnaires have been developed, including the King’s Brief Interstitial Lung Disease questionnaire (K-BILD) [5]. K-BILD is developed and validated for measuring HRQL in a broad range of ILDs [5]. The 15-item K-BILD is easy to complete and considerably shorter than the 50-item SGRQ. In addition, K-BILD has a stronger correlation to pulmonary function tests (PFTs) than SGRQ [5].

K-BILD has been validated in patients with a number of different ILDs. However, no studies have validated K-BILD in a large cohort of patients with IPF [5, 6]. As IPF carries the worst prognosis among ILDs, validation of K-BILD is of great importance in this group of patients. Also, the correlation of quality of life to time since IPF diagnosis has not been reported in other K-BILD studies [5, 6], and the validity of K-BILD in incident compared to prevalent patients is yet uncovered.

Dyspnoea is a central symptom in IPF limiting many daily activities. Thus, dyspnoea is a major determinant of HRQL [7]. To our knowledge, the ability of K-BILD to reflect the impact of dyspnoea on HRQL according to patients with IPF has not been examined; however, it constitutes an important aspect of validity that warrants further investigation.

The 6-min walk test (6MWT) is widely used to evaluate the physical performance of patients with IPF. The test provides valuable information on functional capacity which is not obtained by PFTs, and distance walked during the 6MWT (6MWD) correlates to HRQL [8]. However, the relationship between K-BILD and 6MWD has not been determined in previously published studies.

K-BILD has only been translated from English into a few other languages [6, 9]. Currently, there is no disease-specific HRQL questionnaire for patients with IPF or other ILDs in Danish. When translated into other languages, international use of validated, disease-specific HRQL instruments in clinical trials and daily clinical practice is promoted and will increase awareness of the burdens related to living with IPF. Furthermore, focus on HRQL can promote discussions of palliation at an early stage of IPF, as proposed by the World Health Organization (WHO) [10, 11].

The aim of this study was to validate K-BILD in the, to date, largest cohort of patients with IPF, translate K-BILD into Danish and assess how K-BILD correlates to dyspnoea and 6MWD.

## Methods

### Translation and cultural adaptation

K-BILD was translated into Danish using a multistep forward-backward translation procedure (see Additional file 1) [12, 13]. Subsequently, semi-structured interviews with a focus group of patients were completed to assess the translated version of K-BILD in the target population. During the process, the Danish versions of K-BILD were reviewed by the developers.

### Design

Consecutive patients with IPF were recruited at their outpatient visits at the three Danish tertiary ILD centres at the university hospitals in Aarhus, Odense and Gentofte (Copenhagen). Both incident and prevalent patients were included to increase the generalisability of the results. Patients aged > 18 years with a guideline-based diagnosis of IPF were eligible for inclusion [14, 15]. Patients were excluded, if they were unable to complete the questionnaires due to linguistic or cognitive barriers. A study on the IPF-specific version of SGRQ (SGRQ-I) has been based on the same cohort of patients with IPF [16].

At baseline, the patients completed K-BILD, SGRQ-I, University of California, San Diego Shortness of Breath Questionnaire (SOBQ) and Short Form-36 (SF-36); after 14 days, K-BILD and Global Rating of Change Scales (GRCS) were completed. Questionnaires containing more than 15% missing answers or lacking total or domain scores were excluded from the analyses. At baseline, PFTs (forced vital capacity (FVC) and diffusion capacity of the lung for carbon monoxide (DLCO)) and 6MWT were performed, and gender, age and physiology index (GAP index) was determined [17].

The study was approved by the Central Denmark Region Committee on Health Research Ethics (case no. 1–10–72-87-16) and the Danish Data Protection Agency, and it was registered at clinicaltrials.org (NCT02818712). The participants gave written and informed consent before participating in the study.

### HRQL questionnaires

*K-BILD* is a 15-item self-completed questionnaire measuring HRQL in patients with ILDs [5]. Responses are recorded on a 7-point Likert scale and results in a total score and three domain scores: Psychological, Breathlessness and activities and Chest symptoms. Scores are weighted (logit transformation) and range from 0 to 100, with higher scores indicating better HRQL.

*SGRQ-I* consists of 34 self-completed items assessing HRQL. It was developed as a IPF-specific version of SGRQ and has recently been further validated [16, 18]. Response options vary between several scales. Scores range from 0 to 100 in a total score and three domain scores: Impacts, Activity and Symptoms, higher scores indicate impaired HRQL.

*SOBQ* is a 24-item self-completed questionnaire estimating dyspnoea associated with activities of daily living [19]. Patients score their symptoms on a 6-point scale, and scores range from 0 to 120, higher scores indicate more dyspnoea.

*SF-36* is a generic quality of life questionnaire [20]. It contains 36 self-completed items on a 3–6-point Likert scale, assessing varying aspects of quality of life. Scores range from 0 to 100 and result in eight domain scores and two component scores, higher scores indicate better quality of life.

*GRCS* are self-completed questionnaires designed to assess changes from baseline to the current state of the patients [21]. SGRQ-I was validated in the same cohort of patients, and the items of the domains differ slightly between the two instruments. Therefore, five GRCS were designed for this study; four for the domains of K-BILD (two for breathlessness and activities domain; combined in the analyses) and one for overall HRQL (see Additional file 2 for an English version). Responses are rated on a 11-point Likert scale with numbers ranging from − 5 to 5 and corresponding answers ranging from” Very much worse” over” Unchanged” to” Very much better”.

The questionnaires used in the study were validated in Danish language.

### Validation

*Internal consistency* was evaluated by calculating the interrelatedness of the items in the questionnaire. *Concurrent validity* was evaluated by measuring the correlations of K-BILD to SGRQ-I, SOBQ, SF-36, PFTs and 6MWD. *Test-retest reliability* was evaluated by comparing K-BILD scores at baseline and at 14 days in stable patients. In order to assess the validity of K-BILD in patients with different time since diagnosis, the patients were divided into three subgroups to evaluate the preceding measures. Furthermore, *known groups validity* was evaluated by estimating the ability of K-BILD to distinguish between groups of patients at different stages of the disease. The patients were stratified into “known groups” according to their PFTs (quartiles of FVC and DLCO), 6MWD, use of long-term oxygen therapy (LTOT) and GAP index.

### Statistical analysis

Patients were divided into three subgroups according to the time since diagnosis (TSD): < 1 month, 1–12 months and > 12 months; receiving antifibrotic treatment or not (AFT) and centre of inclusion (CEI).

The characteristics of patients completing or not completing the questionnaires (responders and non-responders) at baseline and after 14 days were compared using Fisher’s exact test for binomial data. Continuous, normally distributed data were analysed using independent two-sample t-test. Otherwise, the Wilcoxon-Mann-Whitney test was used. Normality was assessed by quantile-quantile plots (QQ-plots) and variance homogeneity was accessed using the F-test.

Internal consistency was assessed for K-BILD by calculating Cronbach’s α for each domain and total score; values > 0.7 indicate a reliable internal consistency [22].

Concurrent validity was measured using Pearson’s correlation coefficients, after evaluation of linearity and normality was performed. Correlation coefficients close to 0.7 are considered as strong, close to 0.5 as moderate and close to 0.3 as weak.

Intraclass correlation coefficients (ICC) and Bland-Altman plots were used to examine test-retest reliability. Normality was assessed by QQ-plots. Patients were categorised as stable if they scored − 1 to 1 in GRCS after 14 days. ICC values > 0.7 are considered acceptable measures of reliability [22].

If K-BILD total scores in the known groups followed a normal distribution, the independent two-sample t-test was used for comparison, and otherwise the Wilcoxon-Mann-Whitney test was used. Multiple linear regression analysis was used for comparison of GAP groups. Normality was assessed by QQ-plots, variance homogeneity was accessed using the F-test and the multiple linear regression model was checked by diagnostic plots of the residuals. Effect sizes were calculated from analysis of variance (ANOVA) or multiple linear regression and were reported as partial η^2^: small effect 0.01, medium effect 0.06 and large effect 0.14 [23]. ANOVA was checked by diagnostic plots of the residuals and Bartlett’s test for equal variances. Data were analysed using STATA, version 14.

## Results

### Translation and cultural adaptation

Permission to translate K-BILD was obtained from the developers of the questionnaire [5]. After the forward-backward translation procedure, the Danish version of K-BILD was approved by the developers. Semi-structured interviews were conducted in a representative group of five patients with IPF after completing the Danish version of K-BILD. The developers of K-BILD accepted a minor adjustment after the interviews. The final Danish version of K-BILD can be found in Additional file 3.

### Psychometric validation

A total of 150 patients with IPF were recruited from the three tertiary interstitial lung diseases centres in Denmark (110 patients in Aarhus, 24 in Gentofte and 16 in Odense) between August 2016 and March 2018. Demographics of participants are presented in Table 1.

**Table 1:** Demographics of the participants at inclusion (*n* = 150)

Characteristics	Value
Male (%)	122 (81.3%)
Age, years ± SD	72.9 ± 6.2
Time since diagnosis, years (range)	0.5 (0.0–9.3)
Smoking status	
Current (%)	9 (6.0%)
Former (%)	101 (67.3%)
Never (%)	40 (26.6%)
Long-term oxygen therapy (%)	19 (12.7%)
Antifibrotic treatment	85 (56.7%)
FVC, % predicted ± SD	87.2 ± 23.1
DLCO, % predicted ± SD	48.4 ± 14.1
6MWD, m ± SD	450.3 ± 112.5
K-BILD total ± SD	58.3 ± 12.4
SGRQ-I total ± SD	42.9 ± 22.3
SOBQ ± SD	34.6 ± 25.3
SF-36 PCS ± SD	42.5 ± 8.7
SF-36 MCS ± SD	50.1 ± 10.5

Values are presented as *n* (%), mean ± standard deviation (SD) or median with range [16]. *FVC* Forced vital capacity, *DLCO* Diffusion capacity of the lung for carbon monoxide, *6MWD* Distance walked during the 6-min walk test, *K-BILD* King’s Brief Interstitial Lung Disease questionnaire, *SGRQ-I* IPF-specific version of St. George’s Respiratory Questionnaire, *SOBQ* University of California, San Diego Shortness of Breath Questionnaire, *SF-36* Short Form-36, *PCS* Physical Component Score, *MCS* Mental Component Score

At baseline, the number of questionnaires with more than 15% missing answers, missing domain or total score was: K-BILD 1 (0.7%); SGRQ-I 2 (1.3%); SOBQ 3 (2.0%) and SF-36 1 (0.7%). Eleven patients did not return the questionnaires by mail at 14 days (7.3%). Missing data analyses demonstrated no differences between responders and non-responders, except for the 6MWD where responders at 14 days walked 102.9 m longer on average than non-responders (Additional file 4).

### Internal consistency

Cronbach’s α was high in K-BILD, especially in the psychological domain, the breathlessness and activities domain, and the total score (Table 2). The results of the total score, psychological domain and breathlessness and activities domain were comparable in the TSD, AFT and CEI subgroups (data not shown). Only the results from the chest domain deviated among patients with an IPF diagnosis > 12 months (0.53), patients on antifibrotic treatment (0.61) and patients from Gentofte (0.67) and Odense (0.45).

**Table 2:** Internal consistency of K-BILD

K-BILD	Cronbach’s α
Total	0.92
Psychological	0.93
Breathlessness and activities	0.84
Chest symptoms	0.71

Data represent Cronbach’s α for all patients. *K-BILD* King’s Brief Interstitial Lung Disease questionnaire

### Concurrent validity

K-BILD total and domain scores had moderate to strong correlations to SGRQ-I total and domain scores as well as to SOBQ score. Correlations to SF-36 summary domain scores, PFTs and 6MWD were strong to weak (Table 3). Most correlations were similar for the TSD, AFT and CEI subgroups (data not shown). Exceptions were the weaker correlations between SF-36 MCS and both K-BILD total score and psychological domain in patients with an IPF diagnosis < 1 month (0.37 and 0.29, respectively). Correlations between 6MWD and K-BILD total score and psychological domain became stronger with increasing time since diagnosis (total score: 0.20 to 0.64, psychological domain: 0.13 to 0.46). In patients from Gentofte, K-BILD psychological correlated stronger to SF-36 PCS (0.66) and weaker to SF-36 MCS (0.35). The correlations across most anchors were weaker in the K-BILD psychological domain in patients from Odense (− 0.04 to 0.77).

**Table 3:** Concurrent validity of K-BILD

	SGRQ-I total	SGRQ-I impacts	SGRQ-I activities	SGRQ-I symptoms	SOBQ total	SF-36 PCS	SF-36 MCS	FVC%	DLCO%	6MWD (m)
K-BILD total	− 0.76	− 0.70	− 0.71	− 0.58	− 0.66	0.58	0.56	0.30	0.45	0.43
K-BILD psychological	−0.58	− 0.55	− 0.52	− 0.47	−0.45	0.33	0.60	0.24	0.34	0.31
K-BILD breathlessness and activities	−0.78	− 0.70	−0.76	− 0.57	−0.76	0.69	0.45	0.34	0.50	0.54
K-BILD chest symptoms	−0.69	− 0.66	−0.54	− 0.64	−0.57	0.54	0.44	0.24	0.32	0.30

All data are presented as Pearson’s correlation coefficients for all patients. All correlations had a *p*-value < 0.01 [16]. *K-BILD* King’s Brief Interstitial Lung Disease questionnaire, *SGRQ-I* IPF-specific version of St. George’s Respiratory Questionnaire, *SOBQ* University of California, San Diego Shortness of Breath Questionnaire, *SF-36* Short Form-36, *PCS* Physical Component Score, *MCS* Mental Component Score, *FVC* Forced vital capacity, *DLCO* diffusion capacity of the lung for carbon monoxide, *6MWD* Distance walked during the 6-min walk test

### Test-retest reliability

**Fig. 1:**
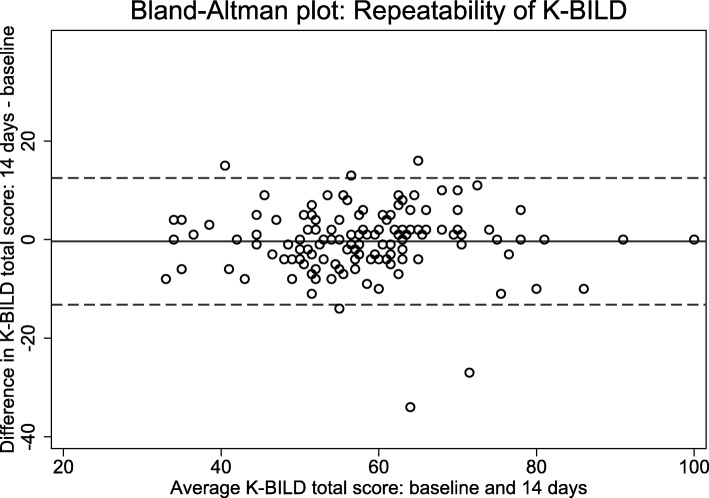
Bland-Altman plot of the repeatability of K-BILD in all responding stable patients. The solid line represents the mean difference, and the dashed lines represent the 95% limits of agreement. *K-BILD*: King’s Brief Interstitial Lung Disease questionnaire

After 14 days, most patients were rated stable as evaluated by GRCS in both overall health status and in the three domains of K-BILD (Table 4). In these patients, K-BILD had high ICC values (Table 4). A Bland-Altman plot showed good agreement between the answers at baseline and after 14 days (Fig. 1). Results were comparable for all TSD, AFT and CEI subgroups (data not shown), except for a slight deviation in the chest domain among patients from Gentofte (0.67).

**Table 4:** Test-retest reliability of K-BILD

K-BILD	*n*	ICC
Total	103 (74.1%)	0.89
Psychological	109 (78.4%)	0.80
Breathlessness and activities	113 (81.3%)	0.89
Chest symptoms	105 (75.5%)	0.76

Data are presented as number of stable patients (% of responders, no. 139) and intraclass correlation coefficients (ICC). *K-BILD* King’s Brief Interstitial Lung Disease questionnaire

### Known groups validity

**Fig. 2:**
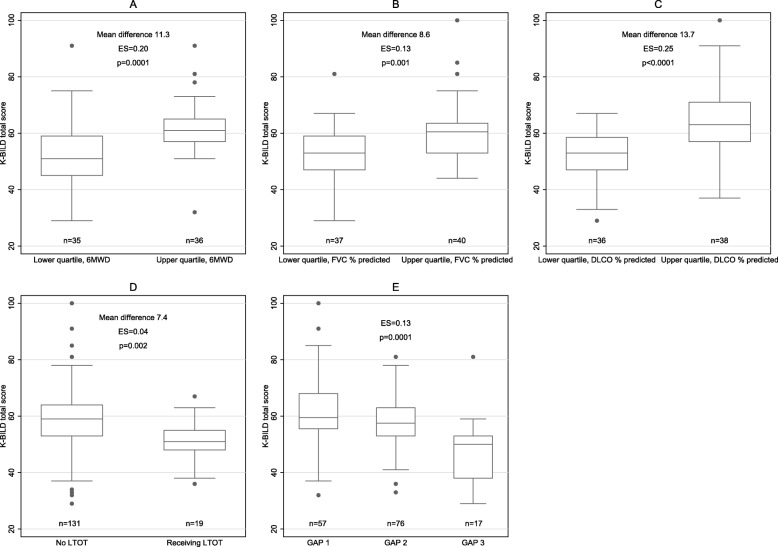
K-BILD total score in (**a**) the lower and upper quartile of 6MWD at baseline, **b** the lower and upper quartile of FVC % predicted at baseline, **c** the lower and upper quartile of DLCO % predicted at baseline, **d** use of long-term oxygen therapy and **e** GAP index. The midlines of the boxes indicate the median values and the boundaries illustrate the 25th and 75th percentiles; the whiskers are the upper (1.5 interquartile range above 75th percentile) and lower adjacent values (1.5 interquartile range below 25th percentile). The dots are outlying values. *6MWD*: Distance walked during the 6-min walk test; *K-BILD*: King’s Brief Interstitial Lung Disease questionnaire. *FVC*: Forced vital capacity; *DLCO*: diffusion capacity of the lung for carbon monoxide; *LTOT*: Long-term oxygen therapy; *GAP*: Gender, age, physiology. *ES*: Effect size (partial η^2^)

Patients in the upper quartiles of 6MWD, FVC % predicted and DLCO % predicted had significantly higher K-BILD total scores than patients in the lower quartiles. Patients receiving LTOT scored significantly lower in K-BILD total score than patients without oxygen therapy. Increasing disease severity according to the GAP index resulted in significantly decreasing K-BILD scores. These findings were supported by strong effect sizes, especially for 6MWD and DLCO, but also for FVC and GAP index (Fig. 2 and Additional file 5).

## Discussion

The current work describes how K-BILD was translated into Danish and validated in the largest cohort of patients with IPF to date. During the translation and cultural adaptation, only minor adjustments were required. The questionnaire was easy to complete for the patients, was shown to be a comprehensive and relevant measure of HRQL and performed well in a non-English speaking population. K-BILD had high internal consistency, moderate to strong concurrent validity, good test-retest reliability, high validity across patients with different time since diagnosis and high discriminative ability in known groups.

This study was conducted among the largest number of patients from a single country included in a translation and validation study of K-BILD. In a previous German study, K-BILD was only translated, but not validated in German [9]. Another validation study included 176 patients, but these were distributed across four countries, with 96 Dutch patients in the largest subgroup [6]. In a larger cohort, results become more valid as this allows for a wider variation in disease severity, views on life and socio-economic backgrounds, providing a better reflection of the patient population.

Additionally, our study included the largest number of measures to evaluate concurrent validity to date, including SOBQ and 6MWD. Even though dyspnoea is a major symptom of IPF and one of the most important factors affecting HRQL [7], no previous studies have compared K-BILD to any specific measures of dyspnoea. SOBQ is a dyspnoea-specific instrument validated for use in IPF [24, 25]. Moderate to strong correlations to SOBQ demonstrate that K-BILD provides a good reflection of this central symptom and indicator of HRQL in IPF. This adds to the validity of K-BILD that can be applied without an additional measure of dyspnoea in a clinical or research setting. Concurrent validity of K-BILD compared to SGRQ-I and SF-36 was moderate to strong. Overall, K-BILD correlated better to other disease-specific questionnaires than to the generic SF-36, which underlines the importance of disease-specific instruments.

6MWD is a functional measure of exercise capacity and decreasing walking distance during the test is associated with declining HRQL [8]. However, no other studies have assessed the relationship between K-BILD and 6MWD. Correlations to 6MWD were moderate to weak with the strongest observations to K-BILD total score and the breathlessness and activity domain. Likewise, correlations to FVC % predicted and DLCO % predicted were moderate to weak. Similar correlations have been reported for other HRQL questionnaires such as A Tool to Assess Quality of life in IPF (ATAQ-IPF), SGRQ and SGRQ-I [3, 18, 26]. This emphasizes that physiological measures of disease severity do not reflect the entire impact of living with IPF. HRQL measures contribute with information about unique aspects of the consequences of the disease that are not otherwise registered and add important information that is not achieved alone by physiological measures of disease severity.

The ability of the instrument to distinguish between patients with different disease severity is also an aspect of validity. K-BILD clearly distinguished patients when grouped into the highest and lowest quartiles of 6MWD, pulmonary function, by GAP index and by using or not using LTOT. Our study is the first to describe this aspect of validity in K-BILD using the 6MWD and GAP index. The discriminative ability of K-BILD in terms of pulmonary function and LTOT has only been reported in one previous study [6].

Internal consistency of K-BILD was good in the chest domain and high in the total score and the two other domains. This indicates a considerable interrelatedness of the items in the questionnaire, meaning that questions measuring the same construct have similar scores. Test-retest reliability was high in K-BILD, confirming that the questionnaire had a good repeatability. Overall, the Danish version of K-BILD performed comparable to the original and translated versions [5, 6].

Even though K-BILD has been developed for use in patients with various ILDs, IPF is the most severe disease with the poorest prognosis. Therefore, validation of K-BILD in patients with IPF is highly important to have a valid instrument to measure HRQL in these patients. Our study has demonstrated that K-BILD is able to reflect the impact on HRQL in patients living with this crippling disease. Increased knowledge about HRQL in patients with IPF may be used to discuss and improve the most cumbersome issues of the disease in daily clinical care. It may also facilitate discussions of palliation as recommended by the WHO at an early stage in progressive diseases [10, 11].

To our knowledge, the validity of K-BILD across patients with different time since diagnosis was evaluated in this study for the first time. As patients with IPF often have respiratory symptoms for a long time before the diagnosis of IPF, the exact disease duration is unknown [27]. Therefore, time since diagnosis was chosen as an average proxy for disease duration. K-BILD proved to be equally valid across several subgroups, increasing the validity and possible application of the instrument to any patient with IPF. However, the chest domain should be interpreted with caution in patients having the diagnosis for more than 12 months, as the internal consistency was moderate for this group. It is possible that the chest symptoms of chest tightness, air hunger and wheezing may change and become less consistent with increasing duration of the disease. The increasing correlations to 6MWD indicate that physical functional capacity has a growing impact on HRQL as the disease advances. The weaker association to SF-36 MCS in incident patients may be incidental or due to a recent serious and life-changing diagnosis, which may influence the general mental health status of SF-36 more than the disease-specific domains of K-BILD. The differing results in patients from Gentofte and Odense may partly be explained by the small sample sizes.

Analyses for missing data showed no significant differences between responders and non-responders in terms of demographics, medical treatment, long-term oxygen therapy or PFTs. The only statistically significant difference was a shorter 6MWD in non-responders compared to responders after 14 days. The results were considered reliable for the entire group of patients, as no significant bias was thought to be introduced due to missing answers.

Compared to other HRQL questionnaires, K-BILD is short with only 15 items and is easy to complete. In comparison, SGRQ consists of 50 items and SGRQ-I contains 34 items [4, 18]. Another IPF-specific HRQL questionnaire is ATAQ-IPF containing 74 items [26]. Despite the shortness of K-BILD, it has the same validity as SGRQ, SGRQ-I and ATAQ-IPF in terms of internal consistency, concurrent validity, test-retest reliability and known groups validity [3, 5, 6, 18, 26]. Another short HRQL measure is the COPD Assessment Test (CAT), which has also been validated in both IPF and other ILDs [28–30]. A short HRQL questionnaire is advantageous in both clinical and research settings for both patients and health care professionals.

As K-BILD has been developed for use in different ILDs, the lack of patients with other ILDs than IPF is a limitation to the Danish version of K-BILD. However, other studies including other ILDs have shown a comparable validity of K-BILD in patients with IPF and patients with other ILDs [5, 6] and it can therefore be assumed that the Danish version of K-BILD will be equally valid in patients with other ILDs. Patients included in the study had relatively well-preserved FVC% and moderately impaired DLCO%. Patients with similar lung function and HRQL have been reported in IPF registries [31, 32]. Preservation of lung function may be due to earlier diagnosis of IPF. Responsiveness and minimal important difference (MID) were not assessed in this study. Patel et al. have reported a MID of 8 unit change (range 6–10) for K-BILD, but the study included only 57 patients, and a larger study of MID is thus needed [33]. The present cohort of patients with IPF participates in a longitudinal study that will evaluate both responsiveness and MID for K-BILD. One of the strengths of our study is the inclusion of the large cohort of patients with IPF with different time since diagnosis. The width of the cohort increases the generalisability of the results to IPF cohorts. Also, the study included new measures of dyspnoea and functional physical capacity that have not been compared to K-BILD before, thus expanding the current knowledge on validity and applicability of K-BILD.

## Conclusions

In conclusion, K-BILD is a valid and reliable instrument to use in patients with IPF and can be applied in patients with different time since diagnosis. The Danish version is as valid and reliable as the original. To a major extent, K-BILD scores reflect the impact of dyspnoea on HRQL and the impact of physical functional capacity measured by 6MWD to a moderate degree. Compared to PFTs alone, K-BILD provides additional information on the burden of living with IPF, and importantly, K-BILD is simple to implement in both research and clinical contexts.

## Supplementary information

**Additional file 1.** Translation process

**Additional file 2.** Global rating of change scales.

**Additional file 3.** Changes and comments in the translation process.

**Additional file 4.** Missing data analyses.

**Additional file 5.** Known groups validity.

## Data Availability

The datasets collected and analysed during the current study are not publicly available due to information that could compromise research participants’ privacy, but are available from the corresponding author on reasonable request.

## Abbreviations

6MWD: Distance walked during the 6-min walk test
ATAQ-IPF: A Tool to Assess Quality of life in IPF
CAT: The COPD Assessment Test
COPD: Chronic obstructive lung disease
DFIS: Dutch/French/Italian/Swedish
DLCO: Diffusion capacity of the lung for carbon monoxide
FVC: Forced vital capacity
GAP: Gender, age and physiology
GRCS: Global Rating of Change Scales
HRQL: Health-related quality of life
ICC: Intraclass correlation coefficients
ILD: Interstitial lung disease
IPF: Idiopathic pulmonary fibrosis
K-BILD: The King’s Brief Interstitial Lung Disease questionnaire
LTOT: Long-term oxygen therapy
MCS: Mental component score
MID: Minimal important difference
PFT: Pulmonary function test
QQ-plots: Quantile-quantile plots
SF-36: The Short Form-36 (SF-36)
SGRQ: The St. Georges Respiratory Questionnaire
SGRQ-I: The IPF-specific version of the St. Georges Respiratory Questionnaire
SOBQ: The University of California, San Diego Shortness of Breath Questionnaire
WHO: World Health Organization
