# Supplementary material for: Validation of the King’s Brief Interstitial Lung Disease questionnaire in Idiopathic Pulmonary Fibrosis
Source: BMC Pulm Med. 2019 Dec 19;19:255. doi: 10.1186/s12890-019-1018-0 (PMC6924069; doi:10.1186/s12890-019-1018-0)
Supplement: Supplementary file 1 — Additional file 1. Translation process [file 12890_2019_1018_MOESM1_ESM.pdf]

Additional files

Additional file 1: Translation process

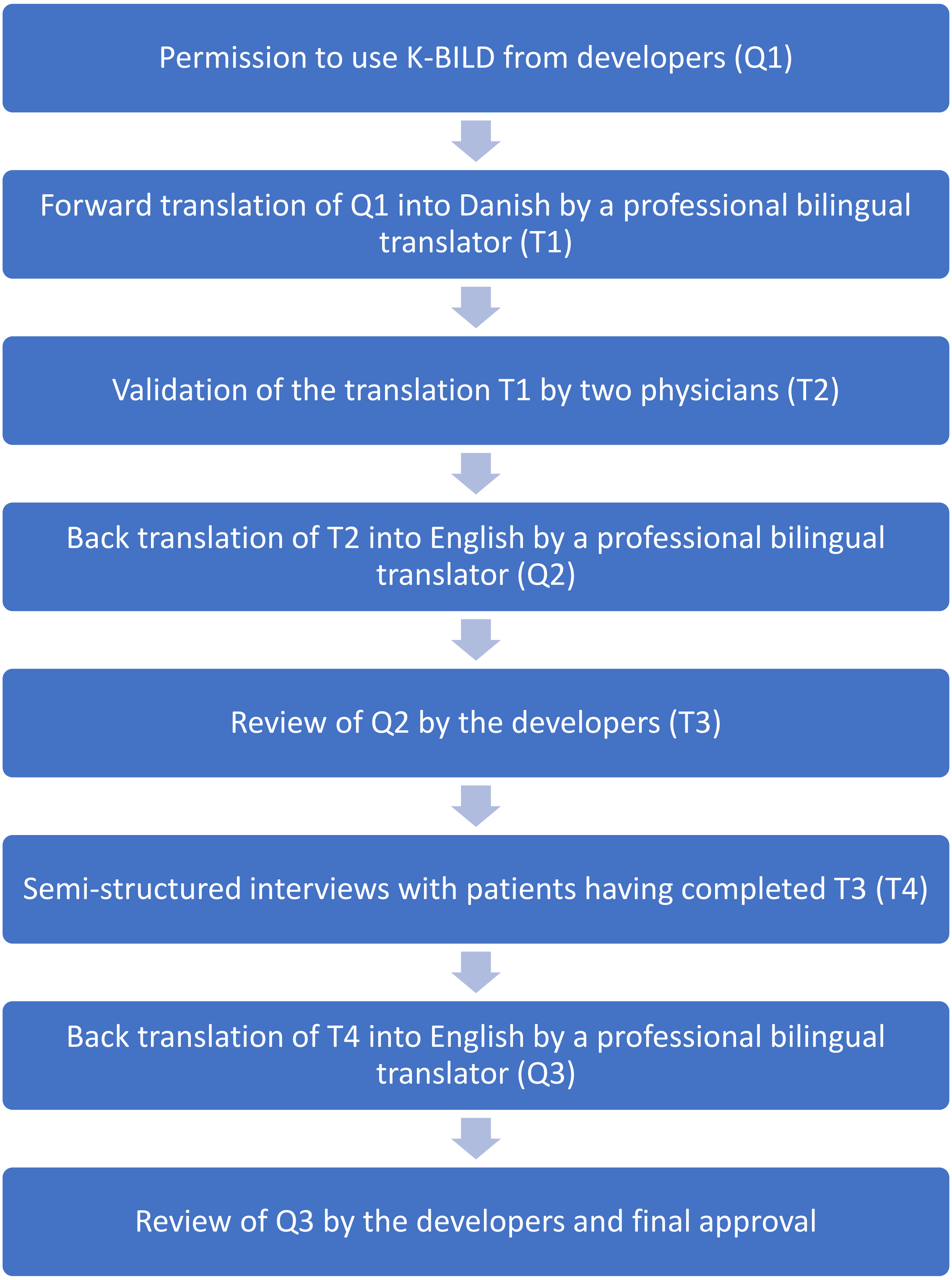

Q: English version of K-BILD, *T:* Translation in Danish
