# Supplementary material for: Validation of the King’s Brief Interstitial Lung Disease questionnaire in Idiopathic Pulmonary Fibrosis
Source: BMC Pulm Med. 2019 Dec 19;19:255. doi: 10.1186/s12890-019-1018-0 (PMC6924069; doi:10.1186/s12890-019-1018-0)
Supplement: Supplementary file 2 — Additional file 2. Global rating of change scales. [file 12890_2019_1018_MOESM2_ESM.pdf]

Additional file 2: Global rating of change scales

Global rating of change scales

These questions concern the impact of your respiratory disease on quality of life and everyday life. Please recall your condition at enrolment in the study. Subsequently, please think about your present condition. Then please reply to the questions concerning whether your condition is better, unchanged or worse.

Name:

Date:

1. Due to your respiratory disease, how would you describe your quality of life today compared to the time of enrolment in the study:

-5	-4	-3	-2	-1	0	1	2	3	4	5
Very much worse					Unchanged					Very much better

2. Due to your respiratory disease, how would you describe your dyspnoea and cough today compared to the time of enrolment in the study:

-5	-4	-3	-2	-1	0	1	2	3	4	5
Very much worse					Unchanged					Very much better

3. Due to your respiratory disease, how would you describe your chest problems today compared to the time of enrolment in the study:

-5	-4	-3	-2	-1	0	1	2	3	4	5
Very much worse					Unchanged					Very much better

4. Due to your respiratory disease, how would you describe your ability to perform your daily activities today compared to the time of enrolment in the study:

-5	-4	-3	-2	-1	0	1	2	3	4	5
Very much worse					Unchanged					Very much better

5. Due to your respiratory disease, how would you describe your social relationships and psychological condition today compared to the time of enrolment in the study:

-5	-4	-3	-2	-1	0	1	2	3	4	5
Very much worse					Unchanged					Very much better
