# Supplementary material for: Validation of the King’s Brief Interstitial Lung Disease questionnaire in Idiopathic Pulmonary Fibrosis
Source: BMC Pulm Med. 2019 Dec 19;19:255. doi: 10.1186/s12890-019-1018-0 (PMC6924069; doi:10.1186/s12890-019-1018-0)
Supplement: Supplementary file 3 — Additional file 3. Changes and comments in the translation process. [file 12890_2019_1018_MOESM3_ESM.pdf]

Additional file 3: Changes and comments in the translation process

T1 -> T2	Question	Changes
	1	"I have been breathless climbing stairs or walking up an incline or hill" changed to "I have lost my breath while climbing stairs or walking uphill"
	4	"make you breathless" changed to "make you lose your breath"
	5	"felt in control of your lung condition" changed to "felt that you have been in control of your lung condition"
	7	"felt the urge to breathe, also known as 'air hunger'" changed to "have felt a strong urge to breathe (hungered for air)"
	8	"my lung condition has made me feel anxious" changed to ", I have felt anxious because of my lung condition"
	9	"experienced 'wheeze' or whistling sounds from your chest" changed to "noticed squeaking or wheezing lung sounds"
	14	"the end of your life" changed to "the last part of our life"

T2 -> T3		No changes

T3 -> T4	Question	Changes
	5	"that you have been in control of your lung condition" changed to "able to cope with your lung condition", as the patients felt that the doctors, and not the patients, were in control

T: Translation in Danish
