# Supplementary material for: Validation of the King’s Brief Interstitial Lung Disease questionnaire in Idiopathic Pulmonary Fibrosis
Source: BMC Pulm Med. 2019 Dec 19;19:255. doi: 10.1186/s12890-019-1018-0 (PMC6924069; doi:10.1186/s12890-019-1018-0)
Supplement: Supplementary file 4 — Additional file 4. Missing data analyses. [file 12890_2019_1018_MOESM4_ESM.pdf]

Additional file 4: Missing data analyses

At baseline

	Responders (*n* = 145)	Non-responders (*n* = 5)	Difference (95% CI)	p-value
**Gender**				
Male, *n*	118	4		1.00
Female, *n*	27	1		
**Age,** years, mean ± SD	72.9 ± 6.3	73.4 ± 3.4	-0.5 (-6.1 to 5.1)	0.86
**Time since diagnosis**, years, median (range)	0.5 (0.0 to 9.3)	0.0 (0.0 to 3.3)		0.26
**Smoking status**				
Current, *n*	8	2		0.12
Former, *n*	99	1		
Never, *n*	38	2		
**Long-term oxygen therapy**				
No, *n*	126	5		1.00
Yes, *n*	19	0		
**Medical treatment for IPF**				
No, *n*	62	3		0.65
Yes, *n*	83	2		
**FVC**, % predicted, mean ± SD	87.2 ± 23.1	88.1 ± 23.2	-1.0 (-21.8 to 19.8)	0.93
**DLCO**, % predicted, mean ± SD	48.5 ± 14.2	47.5 ± 10.7	1.0 (-11.7 to 13.7)	0.88
**6MWT distance**, m, mean ± SD	450.2 ± 113.5	455.3 ± 85.6	-5.0 (118.3 to 108.2)	0.93

Values are presented as *n*, mean ± standard deviation (SD) or median with range(16). *95% CI*: 95% confidence intervals; *IPF*: Idiopathic pulmonary fibrosis; *FVC*: Forced vital capacity; *DLCO*: diffusion capacity of the lung for carbon monoxide; *6MWT*: 6-minute walk test

After 14 days

	**Responders (*n* = 139)**	**Non-responders (*n* = 11)**	**Difference (95% CI)**	**p-value**
**Gender**				
Male, *n*	113	9		1.00
Female, *n*	26	2		
**Age,** years, mean ± SD	72.7 ± 6.2	74.9 ± 7.0	-2.2 (-6.0 to 1.7)	0.27
**Time since diagnosis**, years, median (range)	0.5 (0.0 to 9.3)	0.1 (0.0 to 3.3)		0.38
**Smoking status**				
Current, *n*	7	4		0.09
Former, *n*	96	2		
Never, *n*	36	5		
**Long-term oxygen therapy**				
No, *n*	121	10		1.00
Yes, *n*	18	1		
**Medical treatment for IPF**				
No, *n*	60	5		1.00
Yes, *n*	79	6		
**FVC**, % predicted, mean ± SD	87.2 ± 23.0	87.4 ± 24.5	-0.2 (-14.6 to 14.1)	0.97
**DLCO**, % predicted, mean ± SD	48.6 ± 14.2	46.7 ± 12.7	1.8 (-6.9 to 10.6)	0.68
**6MWT distance**, m, mean ± SD	458.4 ± 107.9	355.5 ± 128.0	102.9 (35.0 to 170.9)	0.003*

Values are presented as *n*, mean ± standard deviation (SD) or median with range. * $p<0.05$. *95% CI*: 95% confidence intervals; *IPF*: Idiopathic pulmonary fibrosis; *FVC*: Forced vital capacity; *DLCO*: diffusion capacity of the lung for carbon monoxide; *6MWT*: 6-minute walk test
