# Supplementary material for: Validation of the King’s Brief Interstitial Lung Disease questionnaire in Idiopathic Pulmonary Fibrosis
Source: BMC Pulm Med. 2019 Dec 19;19:255. doi: 10.1186/s12890-019-1018-0 (PMC6924069; doi:10.1186/s12890-019-1018-0)
Supplement: Supplementary file 5 — Additional file 5. Known groups validity. [file 12890_2019_1018_MOESM5_ESM.pdf]

Additional file 5: Known groups validity

Clinical variable	*n*	Mean K-BILD total score (SD)	Mean difference (95% CI)	p-value	ES
**FVC**					
Lowest quartile	37	52.4 (11.1)	8.6 (3.5 to 13.8)	0.001	0.13
Highest quartile	40	61.0 (11.3)			
**DLCO**					
Lowest quartile	36	52.1 (9.3)	13.7*	<0.0001	0.25
Highest quartile	37	65.8 (2.3)			
**6MWD**					
Lowest quartile	35	51.7 (12.2)	11.3 (5.8 to 16.7)	0.0001	0.20
Highest quartile	36	62.9 (10.9)			
**LTOT**					
No LTOT	130	59.3 (12.7)	7.5*	0.002	0.04
Receiving LTOT	19	51.7 (7.7)			
**GAP index**					
1	56	62.8 (14.5)		0.0001	0.13
2	76	57.2 (8.9)			
3	17	48.4 (12.3)			

*: No 95% CI due to the use of a non-parametric test. *K-BILD*: King's Brief Interstitial Lung Disease questionnaire; *95% CI*: 95% confidence intervals; *ES*: Effect size (partial η^2); *FVC*: Forced vital capacity; *DLCO*: diffusion capacity of the lung for carbon monoxide; *6MWD*: Distance walked during the 6-minute walk test; *LTOT*: Long-term oxygen therapy; *GAP*: Gender, age, physiology
